# Silver Nanocomposites Based on a Peach Shell-Derived Carbon Matrix with Antibacterial Activity

**DOI:** 10.3390/nano16070437

**Published:** 2026-04-01

**Authors:** Aleksandra Stankova, Stela Atanasova-Vladimirova, Bogdan Ranguelov, Georgi Avdeev, Nartzislav Petrov, Maria Todorova, Lyudmila Velkova, Aleksandar Dolashki, Pavlina Dolashka

**Affiliations:** 1 Institute of Organic Chemistry with Center of Phytochemistry, Bulgarian Academy of Sciences, 1113 Sofia, Bulgaria; aleksandra.stankova@orgchm.bas.bg (A.S.); krasimirova_m@yahoo.com (M.T.); lyudmila.velkova@orgchm.bas.bg (L.V.); 2 Center of Competence “Clean Technologies for Sustainable Environment—Water, Waste, Energy for Circular Economy”, 1000 Sofia, Bulgaria; nartzi@abv.bg; 3 Institute of Physical Chemistry “Rostislav Kaishev”, Bulgarian Academy of Sciences, 1113 Sofia, Bulgaria; statanasova@ipc.bas.bg (S.A.-V.); rangelov@ipc.bas.bg (B.R.); g_avdeev@ipc.bas.bg (G.A.)

**Keywords:** peach shells, silver nanocomposite with activated carbon, SEM, EDS, XRD, FT-IR, antibacterial activity

## Abstract

Environmental pollution affects the health of living organisms, provoking the emergence of new diseases and infections. In search of sustainable and effective solutions, this study presents a “green” synthesis of five silver nanocomposites with activated carbon (Ag-NACs) obtained from waste biomass from peach shells. The process is carried out in an aqueous environment and does not use toxic organic solvents. The chemical composition, structural properties and morphology of the synthesized Ag-NACs were characterized using scanning electron microscopy (SEM), energy-dispersive X-ray spectroscopy (EDS), X-ray diffraction (XRD), and Fourier-transform infrared spectroscopy (FT-IR). Comparative analysis under different conditions, including silver concentration (0.5%, 1.5%, 4.0% and 8.0%) and impregnation time (24 and 72 h), identified the samples with 4.0% and 8.0% Ag as optimally structured, showing the strongest antibacterial activity. The results confirmed the synergistic effect and mechanism of the carbon composites, which effectively attract bacterial cells while the silver ions inhibit the growth of various pathogens. This efficacy was demonstrated against both Gram-positive (Gram^+^), *Bacillus subtilis*, *Bacillus spizizenii*, *Staphylococcus aureus*, *Listeria innocua*, and *Enterococcus faecium*, and Gram-negative (Gram^−^) bacterial strains, *Escherichia coli*, *Salmonella typhimurium*, *Salmonella enteritidis*, and *Stenotrophomonas maltophilia*, which highlights the significant potential of Ag-NACs.

## 1. Introduction

The massive environmental pollution resulting from the industrial use of chemicals significantly impacts food safety and human health. Consequently, various methods have been developed to remediate soil and purify the air in public buildings, hospitals, and homes to address this global challenge.

Activated carbon (AC) is one of the most widely utilized adsorbents for water purification, as it is cost-effective, readily available, and simple to operate [1,2,3]. These established adsorbents are produced from various forms of agricultural biomass, which serve as renewable raw materials for the synthesis of carbon nanomaterials due to their high carbon content and widespread availability. Agricultural wastes, such as fruit pits, peanut shells, nut shells, sugarcane bagasse, rice husks, oil palm residues, and sawdust, are produced in millions of tons annually and are effectively utilized for this purpose [4].

Activated carbon is recognized as a highly effective adsorbent for pollutant removal owing to its large specific surface area, well-developed porous structure, rapid adsorption kinetics, and high thermal and chemical stability. Critical parameters, including the type and concentration of the activating agent, the manufacturing process, and the nature of the precursors, significantly influence the porosity and adsorption properties of the final product. Through the appropriate selection of precursors and preparation conditions, carbon adsorbents with superior characteristics can be engineered, enabling the efficient adsorption of a wide range of organic compounds and bacterial nutrients [5]. In its various activated and modified forms, AC possesses a high surface area and a predominantly microporous nature, which facilitates its extensive use in water purification [1,6,7], air filtration [8], heavy metal removal [9], bio-environmental remediation [10], and medical applications [11], as well as in catalysis. Furthermore, carbon materials are widely applied as adsorbents, molecular sieves, catalysts, and catalyst carriers [12].

A significant drawback of AC in medical applications is bacterial accumulation in its pores, which can clog it after excessive microbial growth [13]. To avoid this, hybrid or composite materials have been developed by integrating different biomass-derived carbon structures with other components, such as metals, metal oxides, or polymers, thereby improving their functional properties and environmental utility. Metals such as Fe, Co, Ni and Cu are usually incorporated into the biomass-derived carbon matrix by chemical impregnation or hydrothermal treatments to form metal composites [14,15].

The synthesized metal nanocomposites (MNCs) serve as highly efficient sorbents for the removal of organic and inorganic pollutants due to their high specific surface area, and the located atoms on the surface easily coordinate with other atoms [16]. Advantages of these MNCs include an ordered structure, high specific surface area, light weight, mechanical strength, and excellent electrical and thermal conductivity.

Various metals have been used to produce activated carbon nanocomposites, and after combining almond shell AC with silver (Ag), copper (Cu), and magnesium (Mg), compositions with specific desired characteristics were obtained [17]. The development of Ag-NACs has gained particular importance due to their potent antimicrobial and antiviral properties [18]. Although silver has been used in antimicrobial applications since the 19th century [19], it has been replaced by the development of antibiotics. This has led to an increase in bacterial resistance to antibiotics, which has sparked renewed interest in silver as a potent antimicrobial agent [20].

Several methodologies have been published for the synthesis of AgNPs with different shapes and sizes, including chemical reduction [21], laser ablation [22], electron irradiation [23], reduction with inorganic and organic agents [24], and photochemical methods [25]. Their structural properties are usually determined using SEM, which visualizes the size, shape, and distribution of AgNPs on the carbon substrate, the morphology of which varies significantly depending on the synthesis conditions [26].

The high specific surface area and unique physicochemical properties of AgNPs contribute to their potent cytotoxicity against a wide range of microorganisms [27]. This makes them ideal for applications such as coatings for medical devices, wound dressings, and water filtration. The proposed mechanism of action involves the attachment of AgNPs to the cell membrane, disrupting cell permeability and respiratory functions, leading to microbial death [28]. Furthermore, interaction between AgNPs and sulfur-containing proteins on the cell membrane has been suggested, reducing bacterial viability [29]. DNA replication inactivation is also possible after interaction of silver ion (Ag^+^) with phosphorus residues in DNA, inhibiting essential enzymatic functions [30].

Along with the efficacy of synthesized AgNPs, some drawbacks have been reported, such as the presence of toxic residual ions from silver precursors in the final products. Also, although silver acetate is a suitable precursor, its high cost and other limitations pose challenges [31]. Therefore, optimization of “green” methodologies, especially those using biomass as a sustainable feedstock, is necessary for the production of Ag-NACs for sterile materials.

In the present work, a simple, cost-effective and environmentally friendly synthesis of surface-modified Ag-NACs from peach pit shells is presented. The modified Ag-NACs were characterized using XRD, SEM, and surface area/pore size measurements. Furthermore, its inhibitory activity was evaluated against a wide range of Gram-positive (Gram^+^) and Gram-negative (Gram^−^) bacterial strains.

## 2. Materials and Methods

### 2.1. Preparation of Activated Carbon from Waste Peach Shell Biomass and Synthesis of Ag-NAC

To obtain the carbon adsorbent used in the synthesis of Ag-NAC, a waste raw material from the food industry, peach pit shells, was used. After grinding, the shells are subjected to chemical activation with KOH. A process of impregnation of the base with the shells is carried out after mixing the raw material and KOH in a ratio of 1:1. Carbonization of the material occurs after heating to a temperature of 850–900 °C, holding at the final temperature for 30 min. The resulting carbonate is washed with water until the wash water reaches a neutral pH. Textural characterization was performed *via* N_2_ adsorption at 196 °C using a Quantachrome Autosorb iQ-C-XR/MP apparatus (Quantachrome Inc., Boynton Beach, FL, USA) using DFT following vacuum outgassing at 350 °C. The pore size distribution was determined using the DFT method.

For the preparation of Ag-NACs, AC with particle sizes of 0.25–2.5 mm, obtained by pyrolysis of waste biomass from peach pit shells, was used. The modification of the AC support was carried out by the impregnation method, which included pre-wetting with distilled H_2_O of the AC, followed by mixing with different concentrations of 8.0% AgNO_3_ solution, and obtaining 0.5%, 1.5% and 4.0% and 8.0%, respectively, for 24 and 72 h at 25 °C. The obtained Ag-NACs were dried at 80 °C, then heated to 350 °C for 8 h. After completion of the thermal process, the obtained adsorbents with impregnated Ag^+^ were washed three times with distilled H_2_O, followed by drying.

### 2.2. Characterization of the Synthesized Ag-Nanocomposites with Activated Carbon Support

The physiochemical properties of the synthesized Ag-NACs were characterized using several analytical methods and techniques: scanning electron microscopy combined with energy-dispersive spectroscopy (SEM/EDS; JEOL JSM6390, Japan and Oxford Instruments, Abingdon, UK); X-ray diffraction analysis (XRD; goniometer PW1050, Philips, Almelo, The Netherlands) and FT-IR (Infrared [FT-IR] spectrometer INVENIO-R, Bruker, Karlsruhe, Germany). The morphological study and elemental composition of the obtained nanoparticles were investigated using a scanning electron microscopy, model JEOL IT800SHL, with secondary and back scattered electron detectors in the chamber and in the column of the microscope, as well as two energy-dispersive spectrometers, positioned opposite each other, each with an area of 100 square millimeters.

Obtained five samples of the Ag-NACs containing 0.5%, 1.5% 4.0% and 8.0% Ag^+^ were coated with a thin gold film to avoid sample charging. The surface morphologies of the Ag-NACs obtained under different concentrations of Ag^+^ and after 24 h and 72 h were characterized in multiple fields of view using scanning electron microscopy. Several dozen individual SEM images of each sample were obtained and used to construct Ag-NAC size distribution histograms.

The phase composition of the synthesized Ag-NACs was determined using an Empyrean X-ray diffractometer system (PANalytical, Almelo, The Netherlands), equipped with a copper anode X-ray tube (Cu-Kα radiation). Measurements were conducted at a voltage of 40 kV and a current of 40 mA, covering a range of 10° to 80° 2θ with a step size of 0.03° and an exposure time of 3 s per step. The resulting diffraction patterns were analyzed using HighScore Plus software (https://www.malvernpanalytical.com/en/products/category/software/x-ray-diffraction-software/highscore-with-plus-option, accessed on 4 March 2026) in conjunction with the Inorganic Crystal Structure Database (ICSD).

The chemical bonding between the functional groups on the activated carbon surface and the Ag^+^ in the nanocomposites was analyzed using an INVENIO-R FT-IR spectrometer (Bruker, Karlsruhe, Germany) with a resolution of 2 cm^−1^. All FT-IR spectra were recorded over 120 scans using a diamond crystal IRIS single-reflection attenuated total reflectance (ATR) accessory (PIKE Technologies, Fitchburg, WI, USA).

### 2.3. Evaluation of Antibacterial Activity Assessment

The antibacterial activity of synthesized Ag-NACs with varying Ag^+^ concentrations was evaluated against five Gram^+^ (*B. subtilis* NBIMCC 2353, *B. spizizenii* ATCC 6633, *S. aureus* ATCC 6538, *L. innocua* NBIMCC 8755, and *E. faecium*) and four Gram^−^ (*E. coli* ATCC 8739, *S. enteritidis* NBIMCC 8691, *S. typhimurium* ATCC 14028, and *S. maltophilia* ATCC 17666) bacterial strains. Bacterial growth was performed in Nutrient Broth (NB) and Nutrient Agar (NA) (HiMedia Laboratories Pvt. Limited, WagleIndustrial Area, Thane, MS, India) media, prepared according to the manufacturer’s instructions.

Antibacterial activity was analyzed using the agar-well diffusion method [32]. NA plates were inoculated with overnight bacterial cultures adjusted to a density of 0.5 McFarland. Wells were created using a sterile borer, and 100 µL of each Ag-NAC sample was added to the wells at a final concentration of 6.4 mg/mL. The antibacterial efficacy was evaluated by measuring the diameter of the growth inhibition zones (d, mm) after incubation at 37 °C for 24 h.

Minimum Inhibitory Concentration (MIC) values were determined using the broth microdilution method, following the procedures established by the Clinical and Laboratory Standards Institute’s (CLSI) guidelines. Sterile 96-well plates were filled with 180 µL of NB media and different Ag-NACs at final concentrations of 0, 8, 16, 32, 64, 128, 256, 512, 1024, 2048, and 4096 µg/mL. The wells were inoculated with 20 µL of bacterial suspensions (0.5 McFarland) and incubated on a rotor shaker (220 rpm) at 37 °C for 20 h. MIC values were defined as the lowest Ag-NAC that completely inhibited bacterial growth. Results were further confirmed by measuring optical density at 600 nm using a FLUOstar Omega (BMG LABTECH, Ortenberg, Germany) microplate reader.

The Minimum Bactericidal Concentrations (MBCs) were determined on Nutrient Agar plates and after that 100 µL from each microtiter well showing no visible growth was subcultured onto NA plates after incubation at 37 °C for 24 h, the MBC values were defined as the lowest concentrations at which no bacterial colonies were detected.

All data are expressed as means ± standard deviation (SD). Statistical analyses were performed using one-way ANOVA, with significant differences defined at *p* < 0.05.

## 3. Results

### 3.1. Preparation of Carbon Nanocomposite with Impregnated Silver Ions

#### 3.1.1. Preparation of Carbon Adsorbent for Synthesis of Silver Nanocomposites

**Figure 1:**
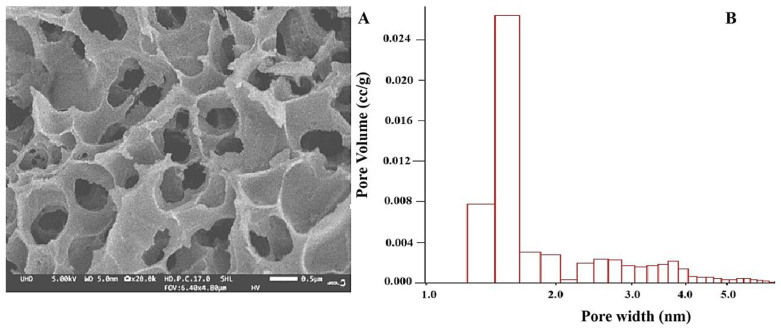
SEM micrograph of: (**A**) output AC from peach shells and (**B**) pore size distribution of an adsorbent obtained by chemical activation of the raw material with KOH.

To obtain nanocomposite with activated carbon impregnated with silver, a carbon matrix obtained from biomass from waste product peach shells was used. This carbon adsorbent is characterized by a particle size of 0.25–2.5 mm and a high BET specific surface area of 1210 m^2^/g, characterized by a complex network of micro-, meso- and macropores (Figure 1A). In accordance with the objectives of the study, the adsorbent was subjected to additional treatment with KOH, as a result of which a high density of oxygen-containing functional groups was achieved on the surface, which serve as centers for the formation of composites with various metal ions (Figure 1B). The analytical data illustrated in Figure 1B show a predominant content of micropores with diameters up to 2 nm and mesopores with sizes from 2 to 50 nm. The adsorption isotherm profile further indicates that the adsorbent contains macropores with sizes greater than 50 nm.

#### 3.1.2. Synthesis of Silver Nanocomposites with Activated Carbon (Ag-NACs)

The main methods that are mainly applied for loading surfaces with metals are by ion exchange and impregnation [33]. The modification of the carbon support from peach shells to silver nanocomposite carbon adsorbents was performed by the basic wet impregnation method. To introduce metals other than the existing C, S, N, and O atoms into the structure and volume of the carbon composite, Ag^+^ were used. Pre-wetted 0.5 g of AC with distilled H_2_O was mixed with different concentrations of an aqueous solution of AgNO_3_, and four Ag-NACs were obtained. The Ag content in the saturated solution was adjusted so that the Ag-NACs contained Ag in a final concentration of Ag-NAC1 (0.5%), Ag-NAC2 (1.5%), Ag-NAC3 (4.0%) and Ag-NAC4 (8.0%).

The as-prepared AC samples, impregnated with varying Ag^+^ concentrations, were homogenized at 150 rpm for 24 h, followed by drying in a forced-air oven at 80 °C for 8 h. The Ag-NACs were stabilized *via* thermal treatment at 360 °C for 8 h; this step facilitates the fixation of Ag^+^ and their partial reduction to nanoparticles onto the carbon matrix. To eliminate impurities, the Ag-NACs were washed three times with deionized water post-thermal treatment and subsequently dried at 80 °C for 24 h. Aqueous impregnation of Ag^+^ onto the carbon matrix for 24 h yielded four distinct samples: Ag-NAC1 (0.5%), Ag-NAC2 (1.5%), Ag-NAC3 (4.0%), and Ag-NAC4 (8.0%). Additionally, the influence of impregnation time was investigated by comparing Ag-NAC4 (24 h) with a sample subjected to an extended 72 h impregnation (Ag-NAC5).

### 3.2. Structural and Morphological Analysis of the Synthesized Ag-NACs

The structural and morphological characteristics of the five Ag-NACs synthesized under different reaction conditions, such as impregnated Ag^+^ concentration from 0.5% to 8.0% and extended impregnation from 24 (Ag-NAC1, Ag-NAC2, Ag-NAC3 and Ag-NAC4) to 72 h (Ag-NAC5), were analyzed using SEM, EDS, XRD and FTIR.

#### 3.2.1. Structural Characterization of Ag-NACs by Scanning Electron Microscopy

**Figure 2:**
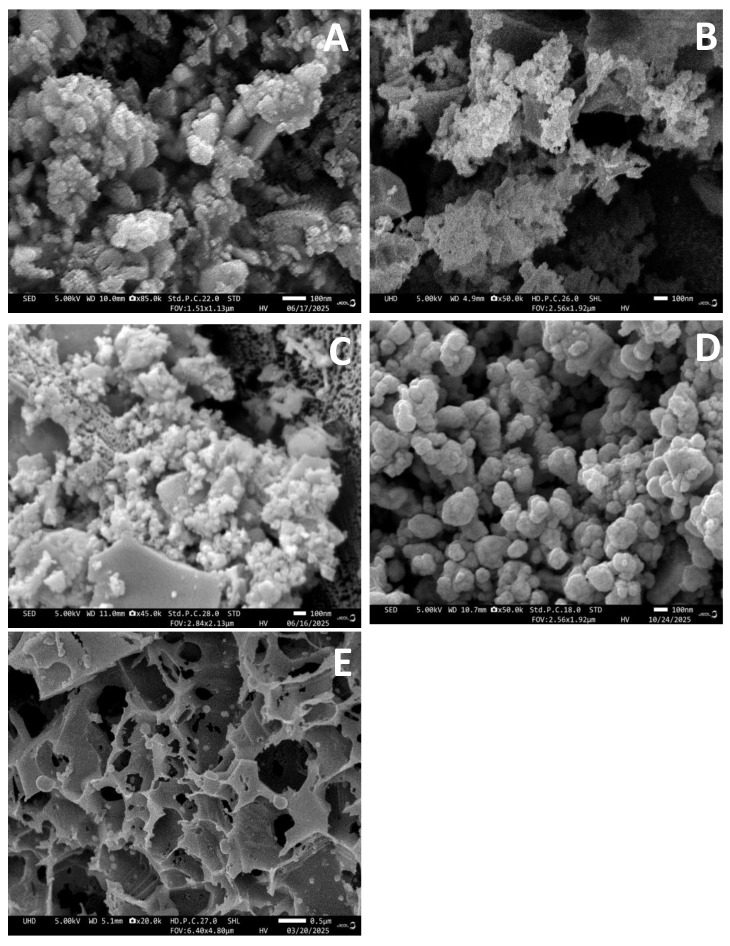
SEM images of Ag-NACs obtained after impregnation of silver ions on carbon adsorbent after 24 h: (**A**) Ag-NAC1 (Sample 1); (**B**) Ag-NAC2 (Sample 2); (**C**) Ag-NAC3 (Sample 3); and (**D**) Ag-NAC4 (Sample 4); and (**E**) Ag-NAC5 (Sample 5) after 72 h of impregnation.

The SEM analysis (Figure 1A) shows that the AgNPs in Ag-NAC1 are mostly spherical in shape. With increasing Ag^+^ concentration in Ag-NAC2 (Figure 1B), the inherent porous structure of AB and irregularly shaped carbon particles is preserved, but a slight “smoothing” is also observed, probably due to the increased metal coverage. In two of the samples, Ag-NAC3 (Figure 2C) and Ag-NAC4 (Figure 2D), numerous microscopic voids were formed, increasing the total surface area of the composite. At a higher concentration of 8.0% Ag, well-defined, almost spherical nanoparticles were obtained, as presented in the SEM image in Figure 2D.

The influence of the impregnation time of Ag^+^ on the AC matrix was evaluated after 24 and 72 h. Comparative analysis between samples Ag-NAC4 and Ag-NAC5, which contain 8.0% Ag^+^, revealed different morphologies of Ag-NACs after 24 h and 72 h of impregnation (Figure 2D and Figure 2E, respectively).

#### 3.2.2. Elemental Composition Analysis of Ag-NACs via Energy-Dispersive X-Ray Spectroscopy

**Figure 3:**
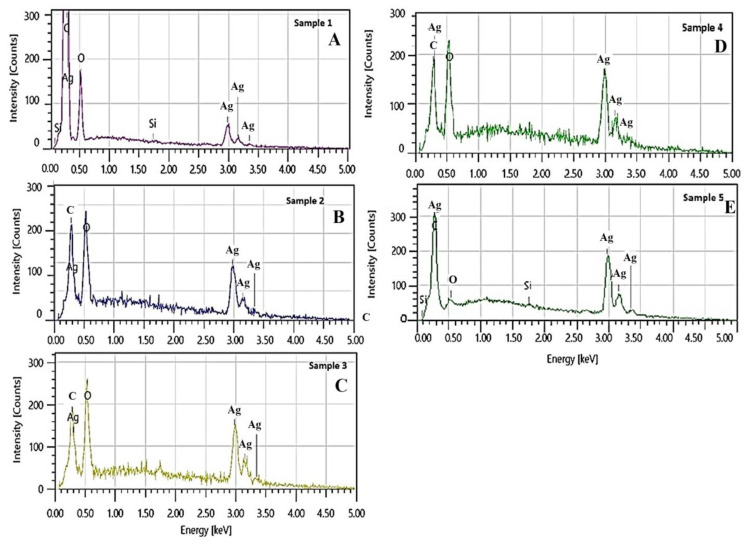
EDS analysis of Ag-NACs: (**A**) Ag-NAC1; (**B**) Ag-NAC2; (**C**) Ag-NAC3; (**D**) Ag-NAC4; and (**E**) Ag-NAC5.

Energy-dispersive X-ray spectra EDS provide further evidence of silver presence in the nanocomposites, with three clearly defined characteristic peaks: a high-intensity peak at 3.00 keV and two lower-intensity peaks at 3.16 keV and 3.5 keV (Figure 3).

Comparative EDS analysis shows that an increasing Ag^+^ after 24 h impregnation leads to a proportional increase in the intensity of the peaks in samples Ag-NAC3 and Ag-NAC4, reflecting the formation of nanoparticles with well-defined shapes and sizes. Furthermore, the impregnation from 24 to 72 h shows the highest peak intensities observed in the sample Ag-NAC5. The elemental composition of the Ag-NAC composites, determined via EDS analysis, is presented in Table 1.

The results indicate that the synthesized Ag-NACs are primarily composed of silver (Ag), oxygen (O), carbon (C), and silicon (Si). The elemental weight ratios shift as the silver loading increases, resulting in a concomitant decrease in the relative concentration of other elements. The highest silver content was measured in sample Ag-NAC4 (96.61% Ag, 1.6% O, and 1.79% C). A comparative analysis between Ag-NAC4 and Ag-NAC5 reveals that the silver content remains nearly constant after 72 h of impregnation (96.49% Ag vs. 96.61% Ag), while the carbon and oxygen levels show slight variations.

#### 3.2.3. Particle Size Distribution Analysis of Ag-NACs

**Figure 4:**
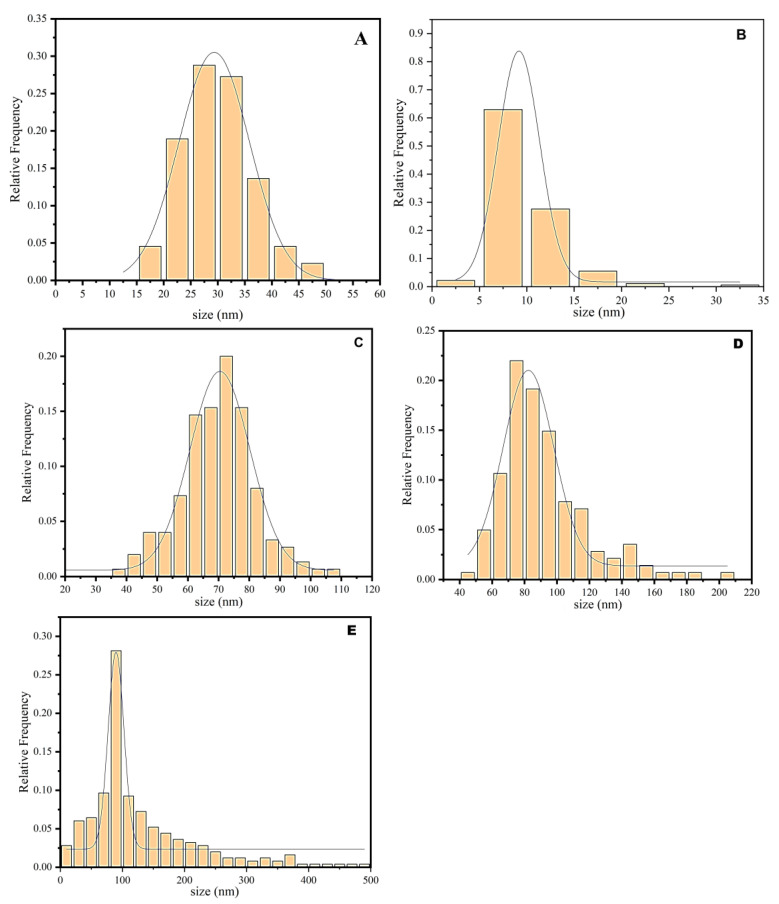
Histograms of particle size distribution in AgNACs and Gaussian-shaped peaks after 24 h of impregnation: (**A**) Ag-NAC1; (**B**) Ag-NAC2; (**C**) Ag-NAC3; (**D**,**E**) after 72 h Ag-NAC5.

Since the particles exhibited a predominantly spherical morphology, their size was determined as the particle diameter measured directly from the 2D projections in the SEM images using the built-in measurement software. The size distribution of the nanoparticles on the alternating current layer was determined from measurements of over 600 individual particles from the SEM images to ensure statistical significance. The size distribution histograms generated for each Ag+ concentration showed well-defined, approximately Gaussian peaks with slight asymmetry (tails), which is typical for nanoparticle systems. They showed a normal distribution and revealed distinct differences between AgNPs obtained at different AgNO_3_ concentrations. Comparative analysis showed that Ag-NAC1 (0.5%) had the narrowest statistical size distribution, with the smallest average sizes expressed by a peak maximum at 28 nm (Figure 4). Approximately 60% of all measured AgNPs fall within the size range of 22 to 32 nm.

It is important to clarify that although SEM measurements are based on 2D projections, the error is considered minimal due to the correct spherical shape of the objects and the low degree of overlap between them. A broader statistical distribution of size on AgNPs in Ag-NAC3, compared to Ag-NAC1 (Figure 4A), expressed with a peak maximum at 70 nm by the histogram for Ag-NAC3 (4.0%) (Figure 4B), shows that approximately 80% of all particles fall within the range of 60 to 80 nm.

The broadest statistical size distribution was recorded for Ag-NAC4 (8.0%), with a peak maximum at 75 nm, with 60% of the particle sizes ranging between 65 and 95 nm (Figure 4C). This distribution also shows a “long tail” extending up to 200 nm, although these larger particles represent a negligible fraction of the total number.

Although the Ag^+^ particles obtained after a prolonged 72 h impregnation period in Ag-NAC5 are relatively evenly distributed over the AC surface (Figure 4E), they differ significantly from the other Ag-NACs in terms of their size distribution (Figure 4).

#### 3.2.4. Characterization of Ag-NACs via X-Ray Diffraction (XRD) Analysis

**Figure 5:**
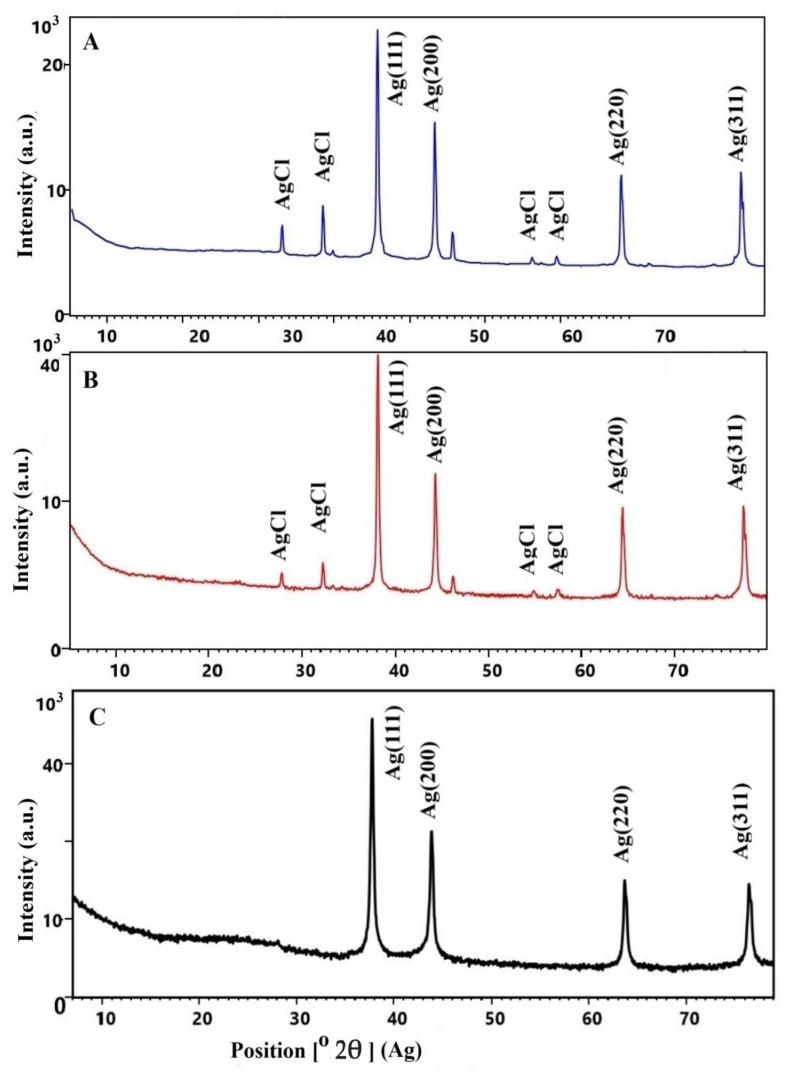
XRD spectra of Ag-NAC, obtained after 24 h of impregnation with different concentrations of AgNO_3_: (**A**) Ag-NAC2 (1.5%); (**B**) Ag-NAC4 (8.0%); and after 72 h of impregnation (**C**) Ag-NAC5 (8.0%) X-ray diffraction.

Additional information for the identification and characterization of Ag-NACs was provided using XRD, another highly sensitive analytical method. The influence of different Ag^+^ concentrations (Ag-NAC2 and Ag-NAC4) and impregnation up to 72 h (Ag-NAC5) was evaluated by analysis of the XRD patterns of the synthesized Ag-NACs (Figure 5). Phase analysis of the Ag-NACs confirmed the formation of AgNPs, which showed excellent agreement with the data from ICSD (card № 98-018-0878). Four characteristic diffraction peaks at 2θ values of 38.0° (111), 44.27° (200), 64.5° (220) and 76.6° (311) were mainly expressed in the diffractograms of the three samples. The intensity of these peaks increases proportionally with increasing Ag^+^ concentration, with the most intense and well-defined peaks observed for Ag-NAC4 (8.0% Ag, Figure 5B) and Ag-NAC5 after 72 h of impregnation (Figure 5C). At lower Ag content, secondary diffraction peaks are also observed in XRD at 2θ = 27.79°, 32.20° and 50–60° (Figure 5A,B). These additional peaks reflect the presence of AgCl (ICSD card № 98-005-6538) in the Ag-NACs.

The phase analysis of the Ag-NAC5 composite obtained after 72 h of impregnation reflects a significant influence of the impregnation time on the synthesis of Ag-NACs. In the diffractogram, only the characteristic peaks of the impregnated AgNPs (ICSD card № 98-018-0878) are observed, without additional peaks corresponding to silver chloride.

X-ray diffraction analysis provides additional important characteristics of Ag-NACs, depending on precursor concentration and impregnation duration, such as the crystallite sizes of the carbon nanocomposites. At lower Ag^+^ concentrations, the crystallite size ranges from 38 to 41 nm (Figure 5A,B). In comparison, at higher concentrations, the crystals grow to 50–54 nm for sample Ag-NAC4 (Figure 5B) and reach 55–67 nm for sample Ag-NAC5 after 72 h of impregnation (Figure 5C).

#### 3.2.5. Functional Group Analysis via Fourier-Transform Infrared Spectroscopy

**Figure 6:**
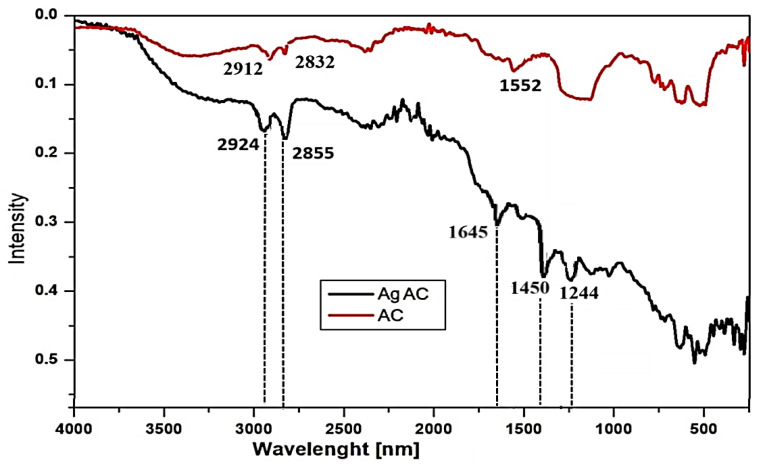
FT-IR spectra of two samples: carbon adsorbent obtained from peach shells (red line), and the synthesized Ag-NACs after 72 h of impregnation—Ag-NAC5 (8.0%) (black line).

The coordination of Ag^+^ with the functional groups of the carbon adsorbent was confirmed using FT-IR spectroscopy. A comparative analysis of the FT-IR spectra of the AC before (red line) and after Ag^+^ loading (black line) is presented in Figure 6.

The broad adsorption bands centered around 3400 cm^−1^ in both spectra are attributed to the O–H stretching vibrations of hydroxyl groups from adsorbed water (hydrogen-bonded hydroxyl groups). Following the incorporation of silver ions, a noticeable shift toward lower wavenumbers is observed in this region. Furthermore, the peaks at 2924 and 2855 cm^−1^, which exhibit higher intensity in the Ag-NACs, confirm the presence of both methylene (–CH_2_–) bridges and aromatic C–H vibrations of aliphatic bonds. One of the most significant observations in the FT-IR analysis of the Ag-NAC is the shift in the C=C stretching vibration associated with the aromatic skeleton.

The carbon matrix is responsible for the weak peak at 1552 cm^−1^, which, in the spectrum of the AC, shifts to a higher wavenumber of 1645 cm^−1^ following the impregnation of Ag^+^ into the AC. The presence of C=C groups in the carbon matrix is further confirmed by the broad band in the 1430–1220 cm^−1^ region; after silver coordination, this band resolves into two well-defined peaks at 1450 cm^−1^ and 1244 cm^−1^ in the Ag-NACs. Additionally, the small peaks appearing at 1244 cm^−1^ and 1058 cm^−1^ are indicative of S=O and C=O stretching vibrations. Evidence for adsorption of Ag on AC is expressed in the changes at 2912 cm^−1^, 2832 cm^−1^, 1552 cm^−1^ and region 1430–1220 cm^−1^.

### 3.3. Antibacterial Activity of Ag-NAC Composites

A well-established property of carbon-based adsorbents is to attract and sequester various bacterial strains without exerting a bactericidal effect. The antibacterial efficacy of Ag-NACs synthesized from peach shells and impregnated with Ag^+^ for 24 h (Ag-NAC1, Ag-NAC2, Ag-NAC3 and Ag-NAC4) and 72 h (Ag-NAC5) was evaluated against a panel of Gram^+^ (*B. subtilis*, *B. spizizenii*, *S. aureus*, *L. innocua*, and *E. faecium*) and Gram^−^ (*E. coli*, *S. typhimurium*, *S. enteritidis*, and *S. maltophilia*) bacterial strains.

#### 3.3.1. Antibacterial Activity of Ag-NACs Against Gram-Positive Bacterial Strains

**Figure 7:**
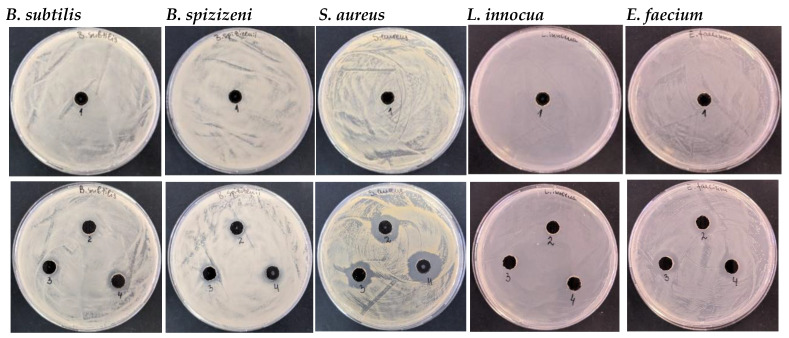
Microbiological analysis of sample number 1 (only AC, line 1) and sample number 2 (Ag-NAC2) and sample number 3 (Ag-NAC4) after 24 h and sample number 4 (Ag-NAC5) after 72 h of impregnation (line 2) against Gram^+^ (*B. subtilis*, *B. spizizenii*, *S. aureus*, *L. innocua* and *E. faecium*).

Initially, the antibacterial efficacy of Ag-NACs synthesized with different AgNP contents was evaluated against a panel of Gram^+^ strains (*B. subtilis*, *B. spizizenii*, *S. aureus*, *L. innocua*, and *E. faecium*). The results summarized in Table 2 show no inhibitory effect at low Ag^+^ concentrations for Ag-NAC1, while Ag-NAC2 exhibits weak inhibitory activity against *B. subtilis* (15 mm) and *S. aureus* (16 mm). A significant broad-spectrum antibacterial effect was observed for Ag-NACs with higher surface loadings of AgNPs, which showed growth inhibition zones ranging from 17 to 20 mm measured against all tested strains (Figure 7, Table 2). The most pronounced antibacterial activity was recorded for Ag-NAC5 against *S. aureus* (20 mm).

#### 3.3.2. Antibacterial Activity of Ag-NACs Against Gram-Negative Bacterial Strains

**Figure 8:**
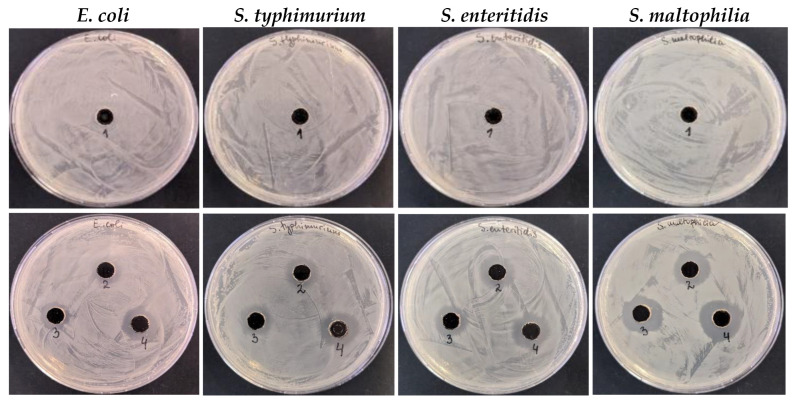
Microbiological analysis of sample number 1 (only AC, line 1) and sample number 2 (Ag-NAC2) and sample number 3 (Ag-NAC4) after 24 h and sample number 4 (Ag-NAC5) after 72 h of impregnation (line 2) against Gram^−^ (*E. coli*, *S. typhimurium*, *S. enteritidis*, *S. maltophilia*) bacterial strains.

The conducted studies also show antibacterial effect of Ag-NACs against Gram^−^ bacterial strains (*E. coli*, *S. typhimurium*, *S. enteritidis*, *S. maltophilia*). The comparative analysis showed a pronounced broad antibacterial activity of the carbon composites with a higher Ag^+^ concentration (Ag-NAC3, Ag-NAC4 and Ag-NAC5) against all tested Gram^−^ bacterial strains *E. coli*, *S. typhimurium*, *S. enteritidis*, *S. maltophilia*. The highest zone of inhibition (17 to 19 mm, respectively) was determined for Ag-NAC4 and Ag-NAC5 against *S. maltophilia* (Table 3, Figure 8).

#### 3.3.3. The Effect of Silver Loading of Different Ag-NACs on Antibacterial Activity Against Gram^−^ and Gram^+^ Strains

To determine the significance of the silver loading of different Ag-NACs and to assess the effect on antibacterial activity against Gram^−^ and Gram^+^ strains, additional comparative analyses were performed on all synthesized nanocomposites using one-way ANOVA (*p* < 0.05). The analysis revealed a statistically significant overall effect between groups (Table 4).

Subsequent post hoc analysis using Tukey HSD did not identify statistically significant differences between individual pairwise comparisons at the critical threshold (q = 3.500) (Table 4 and Table 5). All comparisons showed q-values below the critical value, reflecting the lack of statistical significance between specific pairs of groups.

Notably, comparisons between higher loading levels (Ag-NAC3, Ag-NAC4 and Ag-NAC5) reported minimal mean differences. This suggests stabilization of antibacterial activity at higher concentrations. Tukey’s post hoc analysis showed that the concentrations of 4.0% and 8.0% belonged to the same group with significance (*p* > 0.05), which represented a plateau in efficacy and defined them as optimal loading levels for maximal inhibition.

#### 3.3.4. Determination of MIC and MBC and Antibacterial Efficacy of Ag-NACs

The calculated Minimum Inhibitory Concentration (MIC) and Minimum Bactericidal Concentration (MBC) values of the silver-modified composites provide further insights into the antibacterial efficacy of Ag-NACs against Gram^+^ *(B. subtilis*, *B. spizizenii*, *S. aureus*, *L. innocua*, and *E. faecium*) and Gram^−^ (*E. coli*, *S. typhimurium*, *S. enteritidis*, and *S. maltophilia*) bacterial strains.

**Figure 9:**
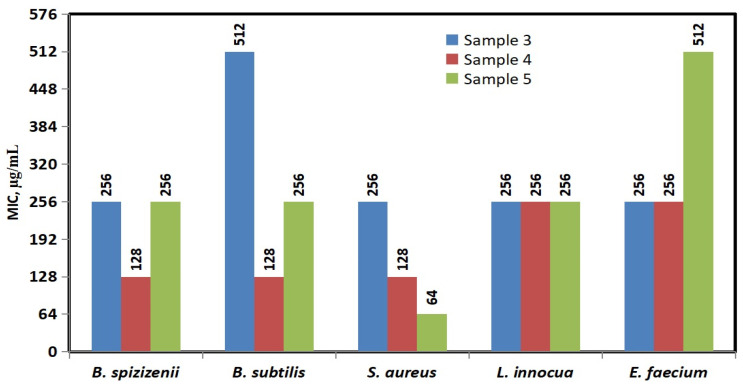
MIC values of Sample 3 (Ag-NAC3) and Sample 4 (Ag-NAC4) after 24 h and Sample 5 (Ag-NAC5) after 72 h of impregnation against Gram^+^ (*B. subtilis*, *B. spizizenii*, *S. aureus*, *L. innocua* and *E. faecium*) bacterial strains.

The comparative MIC analysis presented in Figure 9 highlights that Ag-NAC5 exhibits the lowest MIC value against *S. aureus* (64 µg/mL), followed by Ag-NAC4 against *S. aureus* (128 µg/mL), *B. subtilis* (128 µg/mL), and *B. spizizenii* (128 µg/mL). These results identify them as the most potent inhibitors against these specific bacterial strains.

**Figure 10:**
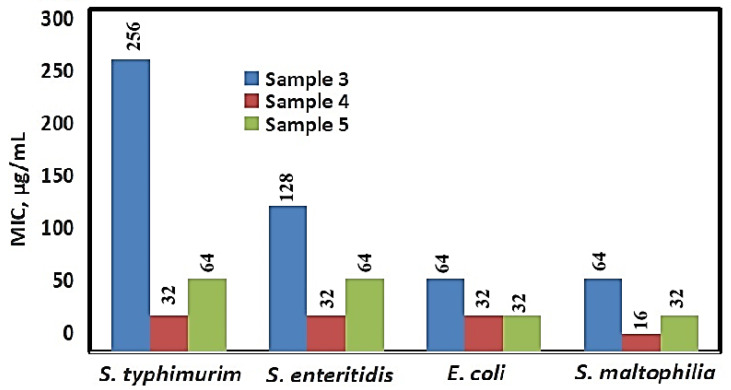
MIC values of Sample 3 (Ag-NAC3) and Sample 4 (Ag-NAC4) after 24 h and Sample 5 (Ag-NAC5) after 72 h of impregnation against Gram^−^ (*E. coli*, *S. typhimurium*, *S. enteritidis*, *S. maltophilia*) bacterial strains.

A significant inhibitory effect on microbial growth was also established for the three Ag-NAC variants against the tested Gram^−^ strains: *E. coli*, *S. typhimurium*, *S. enteritidis*, and *S. maltophilia* (Figure 10). The MIC values for these strains ranged between 16 and 64 µg/mL, with the exception of Ag-NAC3, which exhibited higher MIC values against *S. typhimurium* (256 µg/mL) and *S. enteritidis* (128 µg/mL).

**Figure 11:**
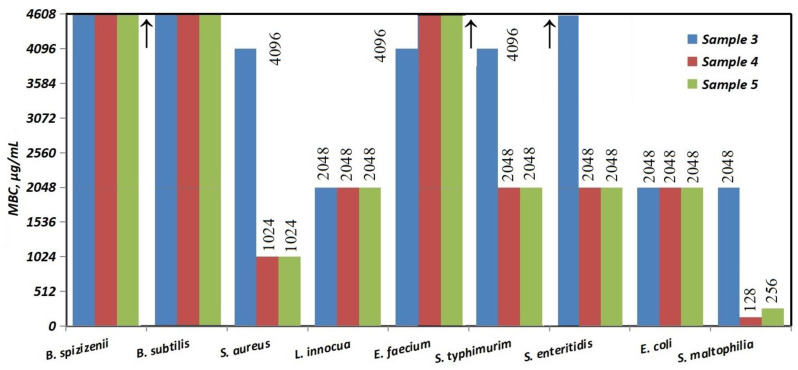
MBC values of Ag-NACs, impregnated for 24 h (Ag-NAC3 and Ag-NAC4) and for 72 h (Ag-NAC5) against Gram^+^ (*B. subtilis*, *B. spizizenii*, *S. aureus*, *L. innocua* and *E. faecium*) and Gram^−^ (*E. coli*, *S. typhimurium*, *S. enteritidis*, *S. maltophilia*) bacterial strains. Arrows—higher values.

The comparison of the Minimum Bactericidal Concentrations (MBCs) in Figure 11 shows high values above 4608 μg/mL for all three Ag-NACs against the two bacterial strains, *B. subtilis* and *B. spizizenii*, as well as for Ag-NAC4 and Ag-NAC5 against *E. faecium* and Ag-NAC3 against *S. enteritidis*. The lowest MBC values, ranging from 128 μg/mL to 2048 μg/mL, were reported for Ag-NAC4 and Ag-NAC5 against Gram^+^ (*S. aureus* and *L. innocua*) and Gram^−^ (*E. coli*, *S. typhimurium*, *S. enteritidis*, *S. maltophilia*) bacterial strains. A significant effect was reported for Ag-NAC4 and Ag-NAC5 against the Gram^−^ strain *S. maltophilia*, with the measured values being, respectively: MIC (16 μg/mL and 32 μg/mL; Figure 10) and MBC (128 μg/mL and 256 μg/mL; Figure 11).

## 4. Discussion

Contaminants in soil, water, and air range from heavy metals and Per- and Polyfluoroalkyl Substances (PFAS) to pathogenic microorganisms, the proliferation of which in sea basins is being enhanced by global warming. Domestic and industrial wastewater often carries bacteria that compromise irrigation systems and drinking water sources. The increasing antibiotic resistance of pathogens, provoked by the discharge of pharmaceutical products into the sewers, is a serious problem. In response to these challenges, various purification methods have been developed, among which the use of carbon adsorbents occupies a central place. They are used as carriers and catalysts and are often produced from millions of tons of agro-industrial wastes—pits, peanut and rice husks, cashew shells, sugar cane, and sawdust [5,34,35,36,37,38]. An innovative and environmentally friendly approach is the photodeposition of silver nanoparticles onto a carbon matrix, a method that uses no harmful reagents and provides promising antiviral and biocidal properties [39].

### 4.1. Synthesis of Carbon Nanocomposite from Peach Shells with Impregnated Silver Ions

Activated carbon is recognized as one of the most effective purification materials due to its high specific surface area, well-developed porous structure and fast adsorption kinetics. The present study presents a synthesized carbon adsorbent from peach shells, combining high porosity with a rich content of functional groups suitable for the synthesis of metal–carbon nanocomposites (MCNs). Peach shells were selected after test studies on various other biomass shells, waste from the Bulgarian canning industry, such as almond shells, cherry pits and plum pits. Peach shell biomass was determined as the most suitable raw material available in Bulgaria for obtaining a carbon adsorbent with the best properties, such as porous texture, large surface area and surface chemical character. A key advantage of the synthesized product is its larger BET surface area (1210 m^2^/g) compared to adsorbents from almond shells (671 m^2^/g) [17] or coconut husks (890 m^2^/g) [10]. This high porosity is a major prerequisite for the excellent capacity of the material to capture toxins and pollutants.

The high adsorption capacity of peach shell AC is a key advantage over alternative adsorbents due to the high density of oxygen-containing functional groups, such as carboxyl, hydroxyl and carbonyl. SEM images reveal the morphology of the material (Figure 1A) after chemical activation with KOH, specific reaction centers are created, suitable for the coordination of metal ions. The adsorption properties of AC are mainly due to the presence of micropores (below 2 nm) but also distributed mesopores (2–50 nm) and macropores (above 50 nm) (Figure 1B). These structural features are critical for the interaction between the carbon matrix and the adsorbate, making the material an ideal substrate for the synthesis of nanoparticles.

Various methodologies for the synthesis of metal–carbon nanocomposites (MCNs) have been described in the literature, but the main techniques include ion exchange, pyrolysis and impregnation [40,41]. An alternative approach is solid-phase synthesis, which facilitates the direct incorporation of heteroatoms into the structure and volume of composites through physical, chemical or combined activation [42]. Physical activation is performed at high temperatures (600–900 °C) with oxidants such as O_2_, water vapor, CO_2_ or ozone [43]. In contrast, chemical activation is performed by thermal treatment (300–500 °C) in common reagents such as phosphoric acid (H_3_PO_4_), bases (NaOH), carbonates (K_2_CO_3_) or salts (ZnCl_2_) [44].

The wet impregnation method is distinguished as a faster procedure, allowing the achievement of higher concentrations of MNPs and ions, localized mainly on the surface of the material [31]. Therefore, in the presented study, five carbon composites were synthesized using this method by varying the Ag^+^ content on a peach shell activated carbon carrier, achieving target concentrations in the final products, respectively: Ag-NAC1 (0.5%), Ag-NAC2 (1.5%), Ag-NAC3 (4.0%) and Ag-NAC4 (8.0%), additionally fixed by heat treatment at 360 °C for 8 h. As a result of the interaction, the AgNPs adhere tightly to the surface of the carbon composite, significantly changing its morphology and adsorption capacity.

### 4.2. Structural and Morphology of the Synthesized Ag-NACs

The successful synthesis and the achieved surface modifications were confirmed by a complex analysis using SEM-EDS, XRD and FT-IR. The chemical nature and surface characteristics of the activated carbon are decisive for the amount of deposited metal ions, which directly affects its catalytic activity. This dependence is confirmed by the SEM images (Figure 1B), showing a progressive accumulation of silver on the surface of Ag-NAC with increasing concentration (from 0.5% to 8.0%). The most pronounced surface clusters of Ag^+^ were registered for Ag-NAC3 (Figure 2B) and Ag-NAC4 (Figure 2D). Another key factor in the development of the surface is the impregnation time, with the comparative analysis showing that increasing the time from 24 h for Ag-NAC4 (Figure 2D) leads to structural improvements in Ag-NAC5 after 72 h (Figure 2E). The presented results confirm the direct influence of these two parameters (concentration and time) on the morphology of the composites, with the initially formed irregular shapes transforming into spherical nanostructures with increasing Ag^+^ concentration and longer exposure time (Figure 2). Optimally shaped composites with the highest potential for adsorption and chemical reactions were found for Ag-NAC5 (72 h of impregnation and 8% Ag^+^; Figure 2E).

The presented results confirm the relationship of another critical parameter—the atomic composition of the synthesized Ag-NACs, with the modification of the activated carbon support. The variation in the elemental composition of the composites is confirmed by the changes in the intensity of the three characteristic peaks at 3.00, 3.16 and 3.5 keV in the comparative EDS analyses presented in Figure 3. The calculated concentrations of the primary elements obtained from the EDS spectra further confirm that the atomic composition of the formed Ag-NAC is directly dependent on the higher content of silver ions.

Unlike the concentrations of Ag^+^, the duration of impregnation does not affect the final elemental composition, which is confirmed by the preservation of the ratio of silver, oxygen, carbon and silicon in the synthesized materials after 24 h of Ag-NAC4 (Ag: 96.61%; O: 1.6%; C: 1.79%) and after 72 h of Ag-NAC5 (Ag: 96.61%; O: 0.38%; C: 3.02%) incubation (Table 1).

The distribution of deposited AgNPs on the surface of AC is another key factor for their activity. The wide range of particle sizes (0.25–2.5 mm) of AC significantly affects the entire process of impregnation of Ag^+^ on AC, from adsorption to final distribution. Ag^+^ reaches the internal active centers of smaller (0.25 mm) particles faster. With large granules (2.5 mm), due to slower diffusion and longer impregnation time, there is a risk of AgNPs agglomeration, which reduces their activity. Therefore, smaller granules will be “oversaturated” with Ag^+^, while larger ones will have only surface-located AgNPs.

Comparative analysis of the histograms prepared based on the SEM images shows a normal (unimodal) distribution and a clear correlation between the Ag^+^ concentration and the size of the resulting particles, with Ag-NAC1 having the smallest average sizes (22–32 nm). The sizes gradually increase, with 80% of the AgNPs in Ag-NAC3 being in the range of 60–80 nm and reaching the range of 65–95 nm for 60% of the particles in Ag-NAC4. An interesting result was observed in Ag-NAC5 (8.0% Ag, 72 h), where relatively uniform impregnation of Ag^+^ was achieved (Figure 3E) and the formation of nanoparticles in a wide range (10–500 nm). Along with the narrow distribution around the maximum of 80–100 nm, a “long tail” of larger particles up to 500 nm is also observed.

The differences found are confirmed by a more pronounced peak in Ag-NAC5 (28% frequency) compared to the other composites (~20%). The differences in the crystal structure and phase of AgNACs were also confirmed by XRD, a well-established method for identifying nanomaterials [45]. Phase analyses of the fractograms (Figure 4) are direct evidence for the synthesis of silver nanoparticles, expressed by sharp and intense peaks at [2θ] = values: 38.0° (111), 44.27° (200), 64.5° (220) and 76.6° (311), with a cubic crystal structure, corresponding to ICSD card № 98-018-0878 and excluding the presence of amorphous forms.

Additional information about the presence of AgCl (ICSD card № 98-005-6538) is provided by the secondary diffraction peaks at 2θ = 27.79°, 32.20° and between 50 and 60° (Figure 4A,B), which decrease in intensity at higher concentrations of impregnated Ag ions on BA. The absence of these peaks in the X-ray diffraction spectrum of Ag-NAC5 (Figure 4C) is evidence of the purity of the formed crystals, which is consistent with the results obtained by other methods.

One of the most important characteristics of the formed Ag-NAC is the approximate size of AgNPs, calculated by the Scherrer formula from the provided XRD analyses. The comparative analysis proves the dependence of the calculated AgHBA3 sizes on the Ag ion concentration, as the smaller Ag-NAC3 size (38 to 41 nm) (Figure 4A) increases at higher Ag ion concentrations and reaches an average Ag-NACs size of 50–54 nm (Figure 4B). Another dependence of the crystal size on the impregnation time is proven, as after 72 h of impregnation of 8.0% silver, Ag-NAC5 with larger sizes in the range of 55–67 nm are formed (Figure 4B).

Information about the specific molecular interactions of AgNPs on the AC surface is provided by the comparative analysis of FT-IR spectra of the starting AC and the formed Ag-NAC5, which include adsorption of Ag ions followed by reduction (Figure 7). The observed change in the position shift and in the intensity of the existing peaks, but not in the appearance of new peaks, reflects changes in coordination or surface adsorption between AC and Ag ions, but not the creation of new covalent bonds. The characteristic changes in the FT-IR spectra of Ag-NACs by shifting the peak position at 1552 cm^−1^ to higher wavenumbers at 1645 cm^−1^ reflect the C=C stretching vibration in the aromatic skeleton, as well as a decrease in the bond strength constant from the electronic influence of the metal, expressed by the change in the broad peak in the region 1430–1220 cm^−1^ into two well-shaped peaks at 1450 and 1244 cm^−1^ of Ag-NAC. Such a change has been published for the peaks 1569 cm^−1^ and 1446 cm^−1^ after adsorption of Ag ions on the surface of AC derived from rice husks, which shift to 1651 cm^−1^ and 1433 cm^−1^, respectively [12]. Other significant changes in the methylene (–CH2–) bridges and aliphatic bonds (C-H) reflected are the shift in the intensity and positions of the peaks at 2912 and 2832 cm^−1^ in the FT-IR spectra of Ag-NACs to positions 2924 and 2855 cm^−1^. All these shifts in the peaks to higher wavenumbers and intensity suggest an increase in rigidity or a change in the electronic distribution of the aromatic rings of AC.

### 4.3. Antibacterial Activity of Ag-NACs Against Gram-Negative and Gram-Positive Bacterial Strains

Various synthesis parameters, such as preparation methods, type and amount of BA, as well as the precursors used, directly affect the porosity and adsorption properties of the obtained five carbon composites with AC matrix from peach shells. The antimicrobial efficacy of the synthesized Ag-NACs depends on the synergy between these structural features and particle size, which allows them to both sequester bacterial cells and effectively inhibit their growth. This hypothesis is supported by the specific activity of Ag-NACs impregnated with different concentrations of Ag^+^ for 24 h (Ag-NAC14) and 72 h (Ag-NAC5) against Gram^+^ strains, *B. subtilis*, *B. spizizenii*, *S. aureus*, *L. innocua* and *E. faecium*, and Gram^−^ strains, *E. coli*, *S. typhimurium*, *S. enteritidis* and *S. maltophilia*.

The antibacterial tests conducted show weak antibacterial activity of the synthesized carbon nanocomposites with a lower concentration of Ag^+^ (Ag-NAC1 and Ag-NAC2); therefore, the antibacterial studies are directed to the carbon nanocomposites with a higher content of Ag^+^ (Ag-NAC3, Ag-NAC4 and Ag-NAC5). The observed significant broad-spectrum effect of Ag-NAC3, Ag-NAC4 and Ag-NAC5 against all tested Gram^+^ strains is likely due to the higher content of AgNPs deposited on the surface, with the most pronounced inhibition observed for Ag-NAC4 and Ag-NAC5 against *S. aureus* and *E. faecium*, with inhibitory zones ranging from 17 to 20 mm (Figure 7, Table 2). These composites showed the lowest MICs against *S. aureus* (64 µg/mL for Ag-NAC5 and 128 µg/mL for Ag-NAC4) and 128 µg/mL for Ag-NAC4 against *B. subtilis* and *B. spizizenii*.

The inhibitory potential of the synthesized Ag-NACs was further validated against Gram^+^ bacterial strains (*E. coli*, *S. typhimurium*, *S. enteritidis* and *S. maltophilia*), with the strongest growth inhibition (17 to 19 mm) achieved by Ag-NAC3, Ag-NAC4 and Ag-NAC5 against *S. maltophilia* (Table 3, Figure 8), with MIC values between 16 and 128 µg/mL (Figure 9). The stronger antibacterial activity of Ag-NAC4 and Ag-NAC5 was also confirmed by the lowest MBCs ranging from 128 µg/mL to 2048 µg/mL (Figure 11) depending on the microorganism. In contrast, a significantly weakened effect was reported for Ag-NAC3, Ag-NAC4 and Ag-NAC5 with high inhibitory and bactericidal doses above 4608 μg/mL for both bacterial strains *B. subtilis* and *B. spizizenii*, as well as for *E. faecium* for samples Ag-NAC4 and Ag-NAC5 and for *S. enteritidis* for Ag-NAC3.

All characteristics such as the measured inhibition zones (Table 2 and Table 3), MIC (Figure 9 and Figure 10) and MBC (Figure 11) demonstrated the most significantly pronounced effect for Ag-NAC4 and Ag-NAC5 against Gram^+^ strain *S. aureus* and Gram^−^ strain *S. maltophilia*.

The obtained results are consistent with published data reporting higher antibacterial activity of AgNACs against Gram^−^ strains, such as *E. coli*, compared to Gram^+^ *S. aureus*. This difference is mainly due to structural differences in their cell walls; the thick peptidoglycan layer of Gram^+^ bacteria acts as a stable protective barrier against various harmful chemical agents [45].

The significance of silver loading for the antibacterial efficacy of the synthesized Ag-NACs was confirmed by statistical one-way analysis of variance (ANOVA). Additional information is provided by the results presented in Table 5 from the post hoc analysis of Turkey. The presented results for Gram^+^ and Gram^−^ microorganisms show that no statistically significant difference (*p* > 0.05) was found between the activity of Ag-NACs with a concentration of 4.0% and 8.0% belonging to the same group, which confirms the achievement of a plateau in efficacy. This pattern supports the tendency to a plateau in activity at higher levels of Ag-NACs.

The effectiveness of the synthesized Ag-NACs with peach shell AC carrier is consistent with published data for other composites, such as Ag-composites with almond shell AC, which, within 24 h, caused 100% microbial reduction in *E. coli* [17], as well as the demonstrated efficacy against several tested pathogens by Ag-NAC from Neem leaves [46] and Ag-NAC from coconut husks with a remarkably low MIC of 16 μg/mL against *E. coli* [10]. Significant antibacterial effects against *E. coli* and *B. subtilis* have been reported for biogenically synthesized AgNAC (by *Enterobacter aerogenes*) and AC from cashew shells [33,34,47]. Other published data show greater tolerance of silver and titanium nanocomposites against *S. aureus* than against *E. coli* [48], as well as of combined Ag and Al_2_O_3_ composites against both Gram^−^ (*E. coli*) and Gram^+^ (*B. subtilis*) bacteria [5].

The antibacterial mechanisms of Ag-NACs are due to a synergistic effect between the carbon matrix and the embedded AgNPs, as the carbon nanocomposite attracts bacterial cells and brings them into direct contact with the active AgNPs. The released Ag^+^ affects multiple vital bacterial functions by adhering to the bacterial cell wall and disrupting the integrity of the cytoplasmic membrane, increasing its permeability [49]. Upon penetration into the cell, Ag+ inhibits the function of key enzymes responsible for ATP synthesis, effectively stopping energy production [50]. They also cause damage to key structural and functional proteins, leading to dysregulation of metabolic processes and eventual cell death [51]. In addition, AgNPs and silver ions interfere with DNA replication and protein synthesis, preventing bacterial proliferation. This multi-targeted approach provides high efficacy and reduces the likelihood of bacteria developing resistance to the composite, increasing the prospect of widespread application in various fields.

## 5. Conclusions

The synthesis of silver–carbon composites from peach shells using an environmentally friendly technology is a promising advance in the fight against pathogens. The presented characteristics of Ag-NACs confirm that after 72 h of impregnation with 8.0% Ag ions on an AC matrix, well-defined, crystalline structures with evenly distributed nanoparticles (55–67 nm) are formed on the surface. The structural properties of the synthesized Ag-NACs explain the higher antibacterial efficacy of Ag-NAC4 and Ag-NAC5 against Gram^−^ *E. coli* compared to Gram^+^ strain *S. aureus*. The most pronounced efficacy of these composites was demonstrated against *S. maltophilia* with inhibitory zones of 19 mm, MIC values (16–32 µg/mL) and MBC (128–256 µg/mL). This inhibitory efficiency is due to the uniform penetration of Ag^+^ ions into the pores of AC and their cytotoxicity, which shows the potential of AgNPs as agents against Gram^+^ and Gram^−^ bacterial strains, even at low silver concentrations.

## Figures and Tables

**Table 1:** EDS analysis of the elemental composition of NAC containing Ag: Ag-NAC1, Ag-NAC2, Ag-NAC3 and Ag-NAC4 after 24 h of impregnation, and Ag-NAC5 after 72 h of impregnation.

Elements	Ag-NAC1		Ag-NAC2		Ag-NAC3		Ag-NAC4		Ag-NAC5	
	Mass, %	Atom, %	Mass, %	Atom, %	Mas, %	Atom, %	Mass, %	Atom, %	Mass, %	Atom, %
C	16.53	59.90	3.24	16.6	2.02	12.36	1.79	13.04	3.02	21.45
O	2.65	7.20	7.19	27.68	5.38	24.67	1.6	8.72	0.38	2.01
Si	0.12	0.34	2.82	6.18	-				0.11	0.33
Ag	80.70	32.56	86.75	49.54	92.6	62.97	96.61	78.24	96.49	76.22
Total	100	100	100	100	100	100	100	100	100	100

**Table 2:** Growth inhibition zones (diameter, mm) of Gram^+^ bacterial strains (*B. subtilis*, *B. spizizenii*, *S. aureus*, *L. innocua*, and *E. faecium*) induced by Ag-NAC composites.

Ag-NACs	Concentration %	*B. subtilis* (D, mm)	*B. spizizenii* (D, mm)	*S. aureus* (D, mm)	*L. innocua* (D, mm)	*B. faecium* (D, mm)
Ag-NAC1	0.5	-	-	-	-	-
Ag-NAC2	1.5	15	-	16	-	-
Ag-NAC3	4.0	12	11	17	10	15
Ag-NAC4	8.0	10	11	16	10	14
Ag-NAC5	8.0	11	11	20	11	14

**Table 3:** Growth inhibition zone (diameter, mm) of Gram^−^ (*E. coli*, *S. typhimurium*, *S. enteritidis*, *S. maltophilia*) bacterial strains by Ag-NACs.

Ag-NAC	Concentration %	*E. coli*	*S. typhimurium*	*S. enteritidis*	*S. maltophilia*
Ag-NAC1	0.5	12	13	-	-
Ag-NAC2	1.5	14	14	-	-
Ag-NAC3	4.0	14	12	12	17
Ag-NAC4	8.0	13	12	12	19
Ag-NAC5	8.0	15	14	13	19

**Table 4:** One-way ANOVA results of Gram^+^ and Gram^−^ bacterial strains.

Source of Variation	SS	df	MS	F	*p*-Value	F Crit
One-way ANOVA results of Gram^+^ bacterial strains						
Between Groups	2489.237	4	622.3092	40.35758	2.5 × 10^−19^	2.472927
Within Groups	1387.789	90	15.41988			
Total	3877.026	94				
One-way ANOVA results of Gram^−^ bacterial strains						
Between Groups	883.7923	4	220.9481	9.125252	7.95 × 10^−6^	2.525215
Within Groups	1452.769	60	24.21282			
Total	2336.562	64				

**Table 5:** Tukey HSD post hoc comparisons of differences in Gram^+^ and Gram^−^ microorganisms.

Comparison	Mean Difference	q-Statistic	q-Critical	Significant
Gram^+^ strains				
Ag-NAC1 vs. Ag-NAC2	6.200	3.033	3.500	No
Ag-NAC1 vs. Ag-NAC3	13.000	6.359	3.500	Yes
Ag-NAC1 vs. Ag-NAC4	13.000	6.359	3.500	Yes
Ag-NAC1 vs. Ag-NAC5	13.400	6.554	3.500	Yes
Ag-NAC2 vs. Ag-NAC3	6.800	3.326	3.500	No
Ag-NAC2 vs. Ag-NAC4	6.800	3.326	3.500	No
Ag-NAC2 vs. Ag-NAC5	7.200	3.522	3.500	Yes
Ag-NAC3 vs. Ag-NAC4	0.000	0.000	3.500	No
Ag-NAC3 vs. Ag-NAC5	0.400	0.196	3.500	No
Ag-NAC4 vs. Ag-NAC5	0.400	0.196	3.500	No
Gram^−^ strains				
Ag-NAC1 vs. Ag-NAC2	0.750	0.282	3.500	No
Ag-NAC1 vs. Ag-NAC3	7.500	2.820	3.500	No
Ag-NAC1 vs. Ag-NAC4	7.750	2.915	3.500	No
Ag-NAC1 vs. Ag-NAC5	9.000	3.385	3.500	No
Ag-NAC2 vs. Ag-NAC3	6.750	2.538	3.500	No
Ag-NAC2 vs. Ag-NAC4	7.000	2.632	3.500	No
Ag-NAC2 vs. Ag-NAC5	8.250	3.103	3.500	No
Ag-NAC3 vs. Ag-NAC4	0.250	0.094	3.500	No
Ag-NAC3 vs. Ag-NAC5	1.500	0.564	3.500	No
Ag-NAC4 vs. Ag-NAC5	1.250	0.470	3.500	No

## Data Availability

The data presented in this study are available on request from the corresponding author.

## Abbreviations

The following Abbreviations are used in this manuscript:

AC	Activated carbon
Ag-NAC	Silver nanocomposites with activated carbon
AgNPs	Silver nanoparticles
EDS	Energy-dispersive X-ray spectroscopy
FT-IR	Fourier-transform infrared spectroscopy
Gram^+^	Gram-positive bacterial strains
Gram^−^	Gram-negative
MCNs	Metal–carbon nanocomposites
MNCs	Metal nanocomposites
PFAS	Per- and Polyfluoroalkyl Substances
SEM	Scanning electron microscopy
XRD	X-ray diffraction
